# Evaluating causal psychological models: A study of language theories of autism using a large sample

**DOI:** 10.3389/fpsyg.2023.1060525

**Published:** 2023-02-24

**Authors:** Bohao Tang, Michael Levine, Jack H. Adamek, Ericka L. Wodka, Brian S. Caffo, Joshua B. Ewen

**Affiliations:** ^1^Bloomberg School of Public Health, Johns Hopkins University, Baltimore, MD, United States; ^2^Kennedy Krieger Institute, Baltimore, MD, United States; ^3^School of Medicine, Johns Hopkins University, Baltimore, MD, United States; ^4^Neurology and Developmental Medicine, Kennedy Krieger Institute, Baltimore, MD, United States

**Keywords:** language, social withdrawal, autism (ASD), psychological theory, large data analysis, causal inference, network analysis

## Abstract

We used a large convenience sample (*n* = 22,223) from the Simons Powering Autism Research (SPARK) dataset to evaluate causal, explanatory theories of core autism symptoms. In particular, the data-items collected supported the testing of theories that posited altered language abilities as cause of social withdrawal, as well as alternative theories that competed with these language theories. Our results using this large dataset converge with the evolution of the field in the decades since these theories were first proposed, namely supporting primary social withdrawal (in some cases of autism) as a cause of altered language development, rather than vice versa.

To accomplish the above empiric goals, we used a highly theory-constrained approach, one which differs from current data-driven modeling trends but is coherent with a very recent resurgence in theory-driven psychology. In addition to careful explication and formalization of theoretical accounts, we propose three principles for future work of this type: specification, quantification, and integration. Specification refers to constraining models with pre-existing data, from both outside and within autism research, with more elaborate models and more veridical measures, and with longitudinal data collection. Quantification refers to using continuous measures of both psychological causes and effects, as well as weighted graphs. This approach avoids “universality and uniqueness” tests that hold that a single cognitive difference could be responsible for a heterogeneous and complex behavioral phenotype. Integration of multiple explanatory paths within a single model helps the field examine for multiple contributors to a single behavioral feature or to multiple behavioral features. It also allows integration of explanatory theories across multiple current-day diagnoses and as well as typical development.

## General introduction

As we approach a century of committed work, how far have we advanced in clarifying the cognitive contributors to the behavioral features of autism in a way that will improve quality of life for autistic individuals? Not nearly as far as we may have hoped. In the current era, many of the great advances in the psychology of autism have tended to be descriptive: reporting that autistic individuals differ in certain capabilities. Yet, for clinicians and advocates, we will maximize our ability to make life-enhancing interventions only when we have a *causal* understanding of how certain cognitive differences produce a wide range of behavioral features that constitute the autism phenotype. If we make a particular modification to the environment, how can we expect it to affect autistic individuals? If we design a new intervention to enhance a particular cognitive ability, what behavioral outcomes are most likely?

Causal theories such as Executive Function (Russell, 1997), Weak Central Coherence (Frith, 1989), Theory of Mind (Baron-Cohen et al., 1985; Rajendran and Mitchell, 2007), Predictive Coding (Courchesne and Allen, 1997; Pellicano and Burr, 2012; Sinha et al., 2014) and primary social accounts (Kanner, 1943; Wing, 1988b; Hobson, 1989; Fletcher-Watson and Happé, 2019) are meant to explain causal relationships rather than merely describing differences with respect to neurotypicals. The concern within psychological science about a movement toward description and away from the proposal and evaluation of causal theories is not unique to the autism literature (Muthukrishna and Henrich, 2019; Fried, 2020; van Rooij and Baggio, 2021); a primary goal of the current work is to refine an approach that shifts focus back to the evaluation of causal accounts.

In biomedical science, the strongest approach to establish causality is with experimenter-controlled perturbation of the system, e.g., the randomized controlled trial (RCT). While there is some progress toward very early interventions that have a corollary benefit of shedding light on causal cognitive mechanisms (Grzadzinski et al., 2021), most of the work that can and will contribute to the evaluation of causal theories in autism is bound to be observational. It is well known that the use of observational data to derive causal conclusions is impacted by known and unknown confounds, yet there are approaches to designing observational tests of causal theories (Rosenbaum, 2017) and statistical approaches (Pearl, 2009, 2018) that can assist in this effort.

When a single theory is under investigation at a time, observational tests of individual causal theories typically rely on falsification: the identification of results that are entailed by the theory and the empiric demonstration through observational data that at least one of these results does not obtain. Because the explanatory power of causal theories depends on their availability to generate accurate predictions over a wide range of samples, measures and experimental contexts (Cronbach and Meehl, 1955; Peirce, 1960; Rosenbaum, 2017), they are inherently susceptible to empirical falsification with observational data.

Such was the case with the psychogenic theories of autism, those accounts that held that disordered parenting was responsible for the development of the autism phenotype. A number of such theories arose during the Psychoanalytic phase of Child Psychiatry (Despert, 1951; Rosenberger and Woolf, 1964; Bettelheim, 1967) and even among early Behavioral Psychology researchers (Ferster, 1961). Thematically similar versions of the theory made somewhat different claims, but the psychogenic family of theories as a whole made several common predictions, and evidence accumulated against these predictions. For example, for the psychogenic accounts to be correct, the parents of autistic children should consistently show greater-than-typical psychopathology (DeMyer, 1975), and, at least as far as Bettelhiem’s version held, children with severe, early social deprivation should exhibit the autism spectrum disorder (ASD) phenotype (Rutter, 1968). Not only one, but *several* of these theoretically entailed predictions were empirically falsified (Rutter, 1968; DeMyer, 1975).

In practice, it is often challenging to falsify a single theory in isolation (Adamek et al., 2022); among other concerns, supporters of a particular theory may “patch” it to reconcile the original claim with disconfirming evidence (Kukla, 2001). Instead, it can be productive to test theories in competition, using contrasts between theories to triangulate specific patters of observations that would provide simultaneous support for one theory and disconfirmation of another (the approach we take here; Pratt, 1971; Kahneman and Klein, 2009) or to examine models quantitatively (“overall model fit” or “relative likelihood”) to determine relative statistical support for one model vs. another (Royall, 1997; Burger et al., 2022). Competitive testing also contributed to the evaluation of psychogenic theories of autism, insofar as the literature of the time informally compared evidence for a psychogenic cause with evidence for a mutually-exclusive “organic” or neurological cause of the condition (DeMyer, 1975). Observations of the presence of neurological signs in autistic individuals, including motor differences and epilepsy (Tuchman and Rapin, 2002), findings which could not be attributed to parenting style, were simultaneously supportive of a neurological account and disconfirming for a psychogenic account.

The goal of the current work was to evaluate several causal theories of key ASD features in competition. This was accomplished by formalizing as statistical models what were originally informal, “verbal” accounts (van Rooij and Blokpoel, 2020). This formalization was accomplished through close reading of the original literature (including the evolution of these theories over time) and the triangulation of areas of theoretical conflict between the different accounts. Our statistical tests then focused on specific areas of identified conflict. The set of theories we used was constrained by data-items available in a convenience dataset.

The theories examined here focus on the role of language in the genesis of broader aspects of the autism phenotype. Because language theories have generally been discarded through the traditional process of evaluation through small samples and dialectic (with a notable degree of graciousness and intellectual honesty on the part of the original proposers), the empirical tests offered below are not likely to offer novel substantive results. However, the benefit of the use of theories well evaluated through traditional means is that we can use their outcome as a sort of reference against which we compare the results of our approach.

Theories that language differences constituted the core cause of a core (albeit heterogenous) aspect of the autism phenotype—namely social withdrawal—was put forth in the 1960’s and 1970’s, by Rutter, Wing and their collaborators. Its origins lay in early observations relating to alterations of low-level auditory perception in autism [Anthony, 1958; Rutter, 1965; *c.f.* more recently (Roberts et al., 2010)]. Explicit comparisons were initially made to the social alterations seen in children with sensory impairments (hearing loss and combined vision-hearing loss; Wing, 1967). Within the milieu of Aphasiology (Geschwind, 1965), both comparisons (Rutter, 1968) and also contrasts began to be made between autism and “developmental aphasia,” now referred to as Developmental Language Disorder (DLD). Social function clearly differed between the two diagnoses, and researchers began to focus on the cognitive underpinnings of language and the use of symbols in particular as being the driving factor in this difference in social function (Wing and Wing, 1971; Churchill, 1972; Ricks and Wing, 1976; Rutter, 1978). Social withdrawal, which was a *sine qua non* in the Kannerian view of autism [Kanner, 1943; Valla et al., 2013; albeit not universal in later conceptualizations (Wing and Gould, 1979)], was proposed at the time to be a consequence of frustrated interpersonal interactions due to the inability of an autistic child to communicate by either verbal or non-verbal means (Rutter, 1966, 1968; Wing and Wing, 1971), while a child with DLD and intact use of symbols could establish relationships using preserved non-verbal communication. Restricted and repetitive behaviors and interests (RRB) were less explicitly explained by these theories than was social function.

Restricted and repetitive behaviors and interests was, however, explained by a theory offered by Hermelin and O’Connor, one which evolved to compete with Wing, Rutter and collaborators’ language theories. Working in the early days of modern Experimental Cognitive Psychology (Neisser, 1967), Hermelin and O'Connor (1970) contended that a broad cognitive difference could explain both RRB and the language/communication phenotype as well as the resulting social behavior, a cognitive difference whose consequences were broader in scope than the symbolic and language differences offered by Wing, Rutter and colleagues. Hermelin and O’Connor’s multiple experimental approaches produced a picture in which autistic children had challenges generating abstract representations of information presented, including but not limited to language information. Several of their tasks involved the serial presentation of stimuli, both linguistic and non-linguistic, and required the participant to guess the next element in a sequence. To do so correctly involved implicitly identifying the rules and relationships among the stimuli presented by the experimenter, so-called “coding.” Children with autism showed difficulty abstracting the implicit relationships among stimuli and predicting the next element over several types of tasks (Hermelin and O'Connor, 1970). These coding differences were held up to explain the symbolic differences proposed by Rutter; restated, differences in symbolism supervene on (i.e., are explained by) differences in coding (Hermelin, 1978).

Due both to the higher cognitive workload imposed by inefficiencies with abstracting and prediction, autistic individuals were understood to take action to simplify their environment and make it more predictable (Belmonte, 2008), in a manner we label as RRB.

Primary-social theories of autism also stood in contrast with language theories. Primary-social theories contended that there is an irreducible and innate social motivation or orientation that is selectively affected in autism. According to the interpretation of some authors (Wing, 1988b; Hobson, 1989), this causal claim goes back to Kanner’s original work (Kanner, 1943). In this account, is a primary difference in degree of social engagement in autism that leads to alterations in the development of language (Bosch, 1962/1970; Richter, 1978)—the direction of the causal association is reversed compared with communication theories. Primary and irreducible social motivation (Wing, 1988b; Hobson, 1989; Chevallier et al., 2012; Keifer et al., 2019) and social orienting (Dawson et al., 1998; Zou, 2004) accounts of autism have re-emerged recently and are the basis for contemporary infant work that focuses on joint attention (Mundy et al., 1986, 1990).

The current work leverages the Simons Foundation Powering Autism Research (SPARK) Database (SPARK Consortium, 2018). This database, which was designed to collect genetic and behavioral (individual−/parent-report) data from 50,000 autistic individuals and their parents, has, at time of the current data release, registered over 71,000 individuals with ASD and a total of over 283,000 individuals (with ASD and parent/siblings) in total.

Our goal was to evaluate language theories of autism alongside the “coding” and primary social theories that developed in explicit competition in the literature, using a single, large dataset. In doing so, we accumulated “lessons learned” for this type of sparse (theoretically constrained) network analysis.

## General methods

### Simons Powering Autism Research (SPARK) dataset, participant selection criteria, and demographics

**Figure 1:**
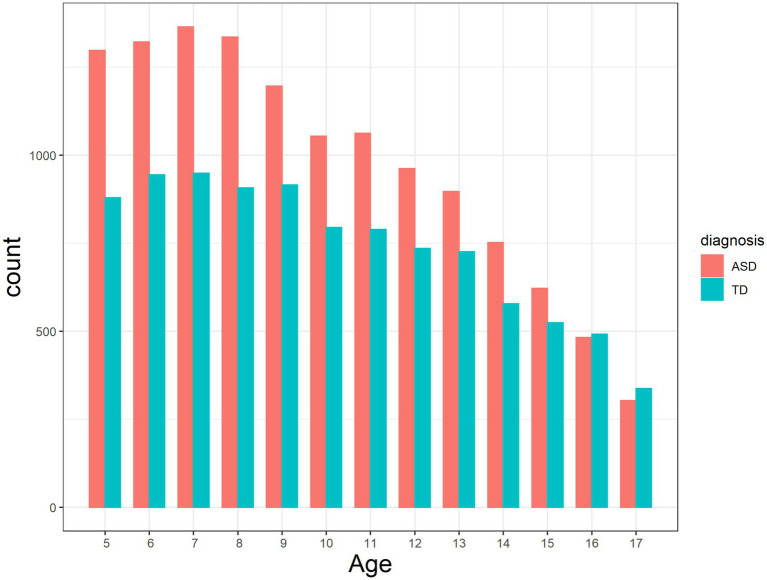
Age distribution by group (“TD” = Sibs group).

We used Release 5 (December 2020) of the SPARK dataset, which included data from a total of 283,520 individuals, of which 94,116 were children (<18 years) diagnosed with ASD or their siblings (“Sibs”). Data cleaning procedures have been described in Dillon et al. (2021). The data were collected under the SPARK protocol (Western Institutional Review Board, Inc.). No identifying data were transmitted to Johns Hopkins Medicine. We included children >5 years of age to have increased confidence in the assessment of language and cognition (Ewen et al., 2019) and because some items referred to performance at age 4–5 years. We further restricted the sample to participants <18 years of age because older individuals completed a somewhat different set of items. We excluded data from all individuals who had missing responses to any item studied. Missingness was believed to be due to task persistence on the part of the family member filling out the instruments and unrelated to the associations studied and not reflective of the effort of the participants themselves. In the end, our dataset included 12,652 children with a parent-reported ASD diagnosis (68% male; 15% also had a parent-reported Intellectual Disability [ID] diagnosis) and 9,571 Sibs (34% male; 1.4% with a parent-reported ID diagnosis). Demographic variables are presented in Table 1. Family of origin was recorded (Family Identity variable), and 4,327 families contributed data on both ASD participants and Sibs (4,495 ASD and 4,469 Sib participants in total). Age distribution is presented in Figure 1. Between-group distributional differences in Age were negligible (Cohen’s *d* = 0.096).

**Table 1:** Demographic variables.

	ASD-only	Sibs-only	ASD+Sibs
Current Age	9.8y (±3.4 [s.d.])	10.1y (±3.5 [s.d.])	9.9y (±3.4 [s.d.])
Age at Diagnosis	5.0y (±2.8 [s.d.])		
Sex	80% male	49% male	67% male
Race			
African American	9.4%	2.0%	6.2%
Asian	3.9%	1.0%	2.7%
Native American	3.7%	0.7%	2.4%
Native Hawaiian/Pacific	0.87%	0.2%	0.6%
White	88.8%	24.2%	61.0%
Other	3.9%	1.6%	3.9%
More than one	20.3%	2.9%	12.8%
Ethnic Hispanic	16.3%	4.8%	11.3%
Family Income			
<$20 k	11.5%	8.5%	10.2%
$21 k-35 k	15.0%	11.6%	13.6%
$36 k-50 k	14.3%	13.3%	13.9%
$51 k-65 k	10.6%	10.3%	10.5%
$66 k-80 k	11.2%	11.4%	11.3%
$81 k-100 k	11.5%	12.5%	11.9%
$101 k-130 k	12.0%	13.2%	12.4%
>$131-160 k	5.3%	6.6%	5.8%
>161 k	8.8%	12.6%	10.4%
Highest Parental Education			
Did not attend high school	0.05%	0.06%	0.05%
Some high school	0.8%	0.7%	0.8%
GED diploma	1.7%	1.3%	1.5%
High school graduate	7.4%	6.7%	7.1%
Trade school	6.4%	5.3%	5.9%
Some college	15.3%	13.1%	14.4%
Associate’s degree	15.2%	14.0%	14.7%
Bachelor’s degree	26.9%	28.3%	27.5%
Graduate/Professional school	26.3%	30.5%	28.0%
Intellectual disability diagnosis	15.4%	1.4%	9.4%
Language disorder diagnosis	47.5%	7.04%	30.1%
Prescribed medication	56.4%	Not reported	

Percentages for Race may not sum to 1 because individual participants may opt out of responding or may select multiple categories.

**Figure 2:**
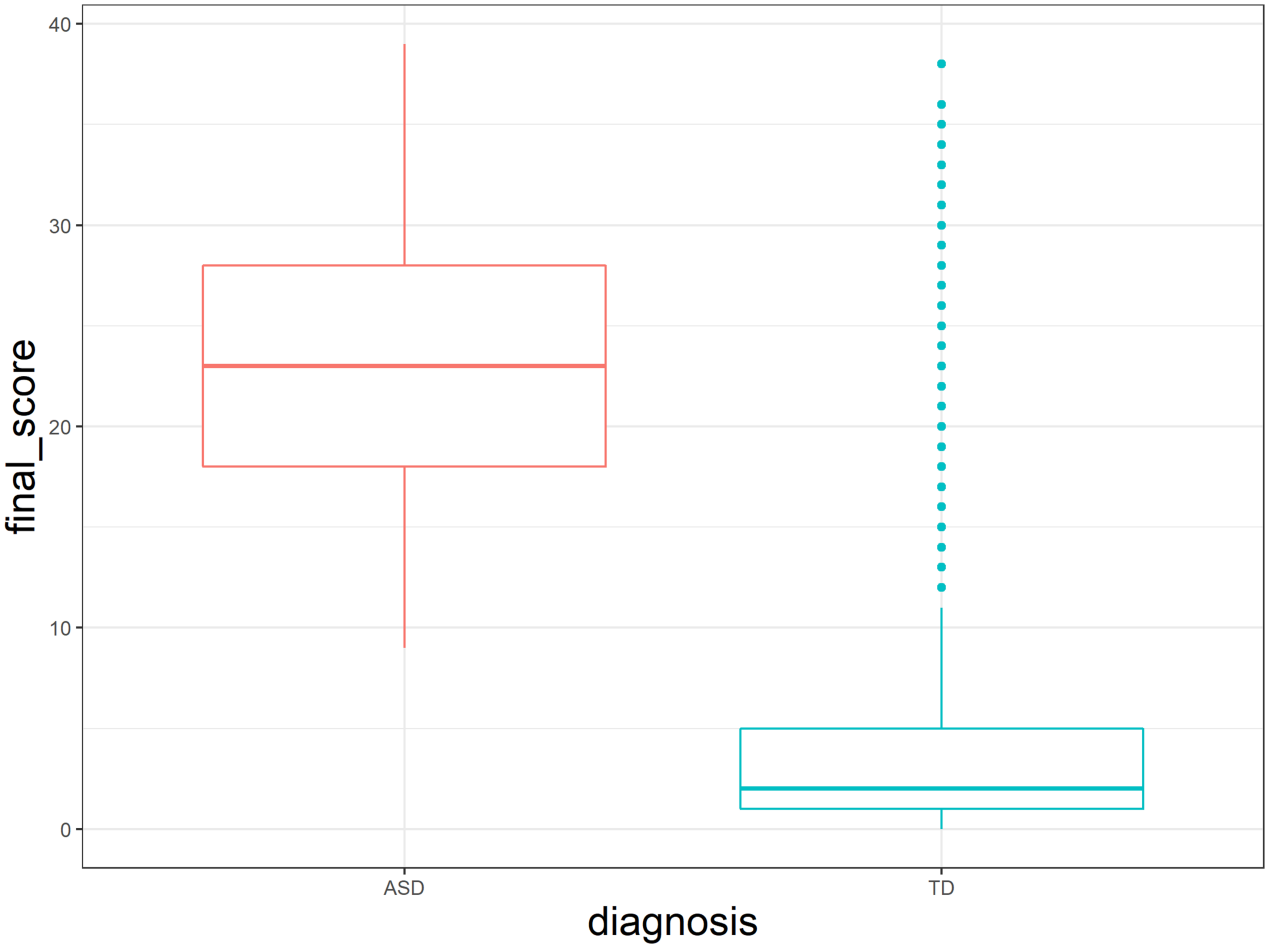
SCQ score distribution over the two samples [ASD and Sibs = “Typically Developing” (TD)].

ASD diagnosis was assessed *via* parent−/caregiver-report, with the explicit instruction that it should be based on a clinical diagnosis (Fombonne et al., 2022). Sex was recorded as parent−/guardian-reported biological sex at birth. Age was recorded at the time that the instrument was completed. Most items were asked in terms of function contemporaneous to time the instrument was filled out, but some items from the Social Communication Questionnaire (SCQ) (Rutter et al., 2003) ask the parent/guardian to recall function at age 4–5 years. SCQ scores for the ASD group were 22.95 ± 6.57 (mean ± SD), range = 9–39; SCQ scores for the TD group were 3.59 ± 4.59 (mean ± SD), range = 0–38 (Figure 2).

### Item selection and psychological construct building and validation

Following literature review around causal theories of key features of ASD, the investigator team reviewed data items collected in this release. We determined that we had sufficient items to evaluate some of the claims regarding communication theories of autism. We used items from the following tables of the SPARK release: Basic Medical Screening, Background History–Child (ASD only), the Background History–Sibling (Sib only), the Repetitive Behaviors Scale–Revised (Lam and Aman, 2007; ASD only), and the SCQ (Rutter et al., 2003). Items related to each theoretical construct, as they were discussed in the literature, were grouped, and we tested construct fit using a confirmatory factor analysis (CFA). We set our criteria as a root mean square of error approximation (RMSEA) < 0.05 (Kim and Mueller, 1978; Brown, 2015) and standardized root mean squared residuals (SRMR) < 0.08 (Kim and Mueller, 1978; Brown, 2015). Any construct that did not initially meet these criteria was split into two factors, and the factor with the more theoretically-relevant items was selected and refit. Each individual participant’s value for each construct was derived from Multivariate Item Response Theory (MIRT), and a normal distribution was imposed on each group. For a few constructs with few items, quantification was obtained *via* Principal Components Analysis (PCA) or as the value of a single item, in cases where only a single item pertained to the construct. Because the ASD and sibling samples included somewhat different items (i.e., the Sibs’ data did not include the RBS-R and some items in the background history), some constructs in the ASD-Only analysis differed in item composition from their corresponding partners in the ASD + Sibs analyzes. All data items present in the Sibs cohort were available in the ASD cohort, but not vice versa. Therefore the constructs in the ASD-Only cohort in some cases derived from a greater number of items than items than corresponding constructs in the Sibs (and therefore ASD + Sibs) cohort. We examined correlations between the “finer” constructs in the ASD-Only sample and the corresponding “coarser” constructs in the ASD + Sibs sample.

### Items in individual constructs

For the *Verbal Language* construct, the ASD + Sibs analyzes included the following items:

- Age in months when first used single words
- Age in months when first combined words into short phrases or sentences with an action word
- Age in months when first combined phrases into longer sentences
- Please select all conditions that you/your child/dependent have/has been diagnosed with by a professional. Please use information included in recent reports from evaluations to help you answer this question.
- Language delay or language disorder

The version used in the ASD-Only analyzes also included the item

- Thinking about child/dependent’s current level of spoken language, would you say it is at or below his/her actual age level? (Above age level | At his/her age level | Slightly below age level | Significantly below age level) The between-group (ASD vs. Sibs) Cohen’s *d* was 0.993, and the correlation between the ASD-Only version of the construct and the ASD + Sibs version of the construct (within the ASD-Only cohort) was *r* = 0.993.

For *Non-Verbal Communication,* both groups used the same items:

- age 4 to 5, normal range of facial expression
- age 4 to 5, spontaneously join and imitate in social games
- age 4 to 5, reciprocal smile
- age 4 to 5, ever integrate eye contact and gesture or and vocalization/speech to request or to direct attention
- age 4 to 5, ever spontaneously point to show
- age 4 to 5, ever spontaneously imitate
- age 4 to 5, shake head no
- facial expression usually appropriate to situation

The between-group (ASD vs. Sibs) Cohen’s *d* was 1.49.

For *Social Withdrawal,* both groups used the same items:

- age 4 to 5, ever want you to join in her/his enjoyment
- age 4 to 5, ever show
- age 4 to 5, ever share
- age 4 to 5, ever chat
- age 4 to 5, interested in children
- age 4 to 5, spontaneously join and imitate in social games
- age 4 to 5, respond positively to child approach
- age 4 to 5, ever join and play cooperatively in games with a group
- age 4 to 5, reciprocal smile
- age 4 to 5, ever comfort
- age 4 to 5, ever spontaneously point to show
- currently have friends or a best friend

The between-group (ASD vs. Sibs) Cohen’s *d* was 2.46. Note that “spontaneously join and imitate” and “spontaneously point to show” were included in both Social Withdrawal and Non-Verbal Communication constructs.

For the *Imaginative Play* construct, both groups (ASD, Sibs) used same items:

- age 4 to 5, ever pretend/make-believe
- age 4 to 5, ever imaginative games with child

Because Imaginative Play relied on only 2 items, individual participants’ value for the construct was assessed *via* Principal Components Analysis (PCA) rather than MIRT. The between-group (ASD vs. TD) Cohen’s *d* was 1.82.

For the *RRB* construct, ASD-Only analyzes used the following items:

- ever special interests, unusual intensity
- ever said the same thing over and over in same way or insisted you say the same thing
- ever interests that preoccupy
- Insists on same routine, household, school/work schedule
- Insists that things take place at specific times
- Insists that things remain in the same place(s)
- Dislikes changes in appearance or behavior of people
- Insists on using a particular door
- Resists changing activities; difficulty with transitions
- arranging/ordering
- Strongly attached to one specific object
- Preoccupation with parts(s) of objects rather than whole
- Fascination, preoccupation with one subject or activity
- checking

The RRB construct in the ASD + Sibs analyzes used only the following constructs:

- ever special interests, unusual intensity
- ever said the same thing over and over in same way or insisted you say the same thing
- ever interests that preoccupy

The between-group Cohen’s *d* was 2.81 for the more limited version of the construct, and the between-version correlation was *r* = 0.38 within the ASD-Only sample.

For the *General Intelligence* construct, the ASD-Only analyzes used the following items:

- When thinking about child/dependent’s general cognitive ability for problem-solving and understanding concepts that do not require language (such as figuring out how things work, or math reasoning): is he/she at or below his/her actual age or grade level?
- Please select all conditions that you/your child/dependent have/has been diagnosed with by a professional. Please use information included in recent reports from evaluations to help you answer this question.
- intellectual disability/cognitive impairment

The ASD + Sibs analyzes used only this items:

- Please select all conditions that you/your child/dependent have/has been diagnosed with by a professional. Please use information included in recent reports from evaluations to help you answer this question.
- intellectual disability/cognitive impairment

The between-group (ASD vs. Sibs) Cohen’s *d* was 0.50, and the between-version correlation was *r* = 0.64 (within the ASD-Only cohort).

*“Symbolism”* was constructed *via* factor analysis of Verbal Language, Non-Verbal Communication and Imaginative Play values. The between-group Cohen’s *d* was 2.10, and the between-version correlation *r* = 0.97.

### Graph building and statistical test selection

Our literature review began with review articles and chapters from multi-author texts (Wing, 1966; Churchill et al., 1971; Rutter, 1971; Wing, 1976a; Rutter and Schopler, 1978; Donnellan, 1985; Wing, 1988a) and monographs (Goldenfarb, 1961; Bosch, 1962/1970; Rimland, 1964; O'gorman, 1967; Hermelin and O'Connor, 1970; Tinbergen and Tinbergen, 1983) and then citations within those works. We identified causal claims related to the role of language differences as causing essential features of autism, as well as contemporaneous claims for abstraction/prediction accounts and social-primary accounts that competed with language theories. We modeled theories as causal graphs with a structure that was consistent with the original authors’ intent as we understood it. Graphs were constructed for those causal claims for which there were adequate items to generate derived constructs in the SPARK dataset. Statistical tests were chosen to evaluate key claims on which the causal model depended, or to highlight areas of explicit contrast between competing claims.

We examined each of the causal theories below using two cohorts: (1) participants diagnosed with ASD only and (2) ASD participants + non-ASD siblings (“Sibs”). Modeling of relationships used Generalized Additive Models (GAM) to account for both linear and second-order effects. Covariates are discussed below; Family Identity was a random effect in all ASD + Sib models. The overall approach to competitive hypothesis testing was “edge-in/edge-out” comparisons rather than overall model fit. Because we accounted for nonlinear effects, the primary metric of effect size was change in variance explained between one model and its contrast (Δ*r*^2^). As the graphs were highly theoretically constrained, some of the methodological concerns associated with data-driven graph-fitting approaches do not apply here, however we have otherwise attempted to comply with recently-published guidelines for network analysis with psychological data (Burger et al., 2022). analyzes were carried out using R version 1.4.3. GAMS were performed using the *mgcv* package (v. 1.8.40), MIRT was using the *mirt* package (v. 1.33.2), and factor analysis using the *lavaan* package (v. 0.6–9).

We covaried for and examined the moderating effect of several potentially confounding variables, to account for alternative hypotheses that were not explicated in the literature; our theoretical justification for each is described in the subsections that follow immediately below. Covariates included, for both cohorts, Age, Sex, and Highest Parental Education (“Parental Education”).^1^ Within the ASD-Only cohort, we also examined for the effect of Family Type (simplex/multiplex). We had additional interest in the impact of DLD diagnosis within the ASD-Only cohort, and ASD diagnosis within the ASD + Sibs cohort. The statistical approach was as follows: as our primary outcome, we contrasted (Δ*r*^2^) a covariates-only GAM and a GAM that contained the covariates plus the theoretically specified predictors. To assess the potential moderating impact of DLD Diagnosis within the ASD-Only group, we next ran a model that also included a DLD Diagnosis covariate and DLD interaction terms with all predictors and all other covariates. In the ASD + Sibs cohort, we examined the moderating effect of ASD Diagnosis *via* a model that included an ASD Diagnosis covariate, ASD Diagnosis interaction terms (with all other predictors and covariates, including DLD diagnosis).

### Moderating effect of age

This convenience sample did not contain longitudinal data, which is a limitation given that some of the theories as proposed were developmental (particularly Models 1 and 4; Happé, 2001), in the sense that they posited that a certain level of function in the “causally upstream” domain would cascade over time and eventually result in a change in relative performance in the “causally downstream” domain. That is to say that there should be a time-lag between the value of a “upstream” construct and the effect on the value of a “downstream” construct. However, this theorized lag would likely result in a moderating effect of Age (assuming our sampling from 5–18 years captures the developmentally relevant ages at which the lag occurs, and with adequate temporal granularity).

### Moderating effect of sex

Although the field is at an early stage of understanding sex differences in autism, there is considerable evidence that they exist in a variety of biological and cognitive domains (Ferri et al., 2018). Moreover, there are known sex differences in language development, even if they are not as great as previously believed (Etchell et al., 2018).

### Moderating effect of parental education

Parental education is known to affect both language development (Roberts et al., 1999) and social development (Hediger et al., 2002), and therefore may confound hypothesized relationships between the two.

### Moderating effect of family type (simplex/multiplex)

As the field sorts out genetic (Bai et al., 2019) and environmental interactions that contribute to the development of the autism behavioral phenotype, it has been hypothesized that the cognitive architecture of autism could differ in affected individuals from families with multiple relatives having autism (“multiplex”) vs. autistic individuals who have no close relatives with the condition (“simplex”; Oerlemans et al., 2016).

### Moderating effect of autism spectrum disorder diagnosis

In most of the models tested here, the original authors used social withdrawal as the behavioral explanandum (“that which must be explained,” i.e., the behavioral consequence of whichever cognitive mechanism is being examined). While we currently understand that social withdrawal is not a universal feature of individuals who carry an ASD diagnosis (Wing and Gould, 1979; Wing, 1996; Phillips et al., 2019; Neuhaus et al., 2021), the theories we are evaluating tended to conflate social withdrawal with what is now viewed as a broad range of autistic social-communication and social-interaction differences. Because autism encompasses a wide and heterogeneous range of cognitive and behavioral differences, it is possible that unmodeled cognitive and behavioral characteristics could confound the relationships that are explicitly modeled. Within the analyzes of the ASD + Sibs cohort, we therefore measured the moderating effect of diagnosis (i.e., the unmodeled autism-related features).

### Moderating effects of developmental language disorder diagnosis

Diagnostic criteria for autism and ASD have changed from the time that the theories studied here were first proposed to the time when SPARK participants enrolled (Rosen et al., 2021). Relevant to the current purposes, the role of language in autism diagnosis has changed over this time. In prior decades, language differences were a necessary diagnostic criterion of the condition (Wing and Gould, 1979). However, more inclusive diagnostic criteria, including those for Asperger syndrome in Diagnostic and Statistical Manual of Mental Disorders (DSM-IV) (American Psychiatric Association, 2000), diagnosed individuals who did not experience significant language delays; the current version of the DSM does not include language differences as a diagnostic criterion (American Psychiatric Association, 2013; Rosen et al., 2021). This change over history raises the concern that the range of language performance of individuals sampled in the SPARK dataset is broader than the range of individuals being described by the authors of the theories studied herein.

Primary conclusions were made on the models without accounting for the DLD diagnosis, as covarying for DLD would regress out theory-targeting language ability from the model, potentially resulting an underestimation of the link between language and social withdrawal, thus increasing risk for spurious falsification. However, to examine for potential biases caused by sampling on a population that has a broader range of language performance than studied by the original theorists, we secondarily measured DLD moderation effects.

## Model 1: Altered language and social withdrawal

### Introduction

Early descriptive observations comparing DLD and autism in children found that children with autism had alterations in both verbal language and non-verbal communication (Rutter et al., 1971; Rutter, 1978). Multiple groups hypothesized that social withdrawal typical of autism might be a direct consequence of these pervasive challenges in communicative ability: “…the poverty of social contact follows from the profound impairment in the development of all forms of language and therefore to the lack of tools necessary for interpersonal communication” (Wing and Wing, 1971); “…language disorder in autism is the primary abnormality…and not one which is secondary to social withdrawal” (Rutter, 1968); “…aphasia in itself is sufficient to account for the marked aloofness and lack of warmth shown by the [autistic] child” (expressed in the language of the time by one of the greatest contributors to the understanding of autism; Rutter, 1966).

Such a developmental relationship seems plausible, even in the current day. Consider the example of the acquired aphasia syndrome of Landau–Kleffner (LKS), in which nocturnal epileptiform activity causes a receptive aphasia that, in some cases, appears to result in secondary social withdrawal (Besag and Vasey, 2019).

**Figure:**
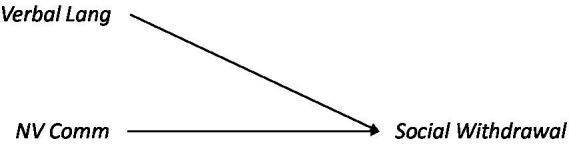


We were conscious of the fact that the items in the Non-Verbal Communication construct assess, in part, the *communicative intent* and *tendency* to produce gestures and other types of non-verbal behaviors, and not solely the *ability* to do so (This fact contrasts with the Verbal Language construct, whose items target language ability rather than communicative use of that ability). Given the sensitivity of Non-Verbal communicative construct to communicative intent, we were concerned for conceptual overlap between Non-Verbal Communication and Social Withdrawal.

### Methods

An efficient way to falsify a *directed* association between communicative performance and social withdrawal is to demonstrate the absence of the corresponding undirected association (Pearl, 2018). We used a GAM to estimate the independent and joint associations between Non-Verbal Communication and Verbal Language (predictors) with Social Withdrawal (outcome), and we compared a covariate-only model with a model also including Non-Verbal Communication and Verbal Language predictors. Covariates were included, and moderating effects were estimated as defined in the General Methods. We also estimated the moderating effect size of General Intelligence in both cohorts.

### Results

In our sample, 47.5% of children with ASD also reported a Language Disorder diagnosis; 7.0% of Sibs did. In the ASD-Only model, the covariates alone explained 7.1% of the variance in Social Withdrawal. There were additional statistically significant marginal associations between Verbal Language and Social Withdrawal (Δ*r*^2^ = 6.5%; *p* < 10^−10^ compared with the covariates-only model) and between Non-Verbal Communication and Social Withdrawal (Δ*r*^2^ = 19.7%; *p* < 10^−10^, compared with the covariates-only model). Verbal Language and Non-Verbal Communication jointly predicted 23.0% of the variance in Social Withdrawal.

Some interaction terms including DLD were statistically significant (DLD × intercept and DLD × Verbal Language), but the overall model fit did not change when DLD covariate and interaction terms were added to the model. For example, the *r*^2^ changed by less than 1 percentage point. The General Intelligence moderation effect increased the model *r*^2^ by 1.1 percentage points.

In the combined ASD + Sibs cohort, after adjusting for covariates, there were statistically significant marginal associations between Verbal Language and Social Withdrawal (Δ*r*^2^ = 19.1%, *p* < 10^−6^ compared with covariates-only model) and between Non-Verbal Communication and Social Withdrawal (Δ*r*^2^ = 45.4%, *p* < 10^−6^) compared with covariates only model. Together, Verbal Language and Non-Verbal Communication jointly predicted 49.5% of the variance in Social Withdrawal.

Developmental language disorder, as a covariate as well as a moderating effect, contributed <1 percentage point to the total variance. The General Intelligence moderation effect increased the model *r*^2^ by 2.3 percentage points.

Several other interaction effects among predictors and covariates were statistically significant, however, their effect sizes were negligible, representing a less than 1 percentage point difference in *r*^2^ between models where the interaction terms were included versus those where they were excluded.

The ASD diagnostic interaction effect was significant (*p* < 10^−6^), and the Δ*r*^2^ (including vs. dropping diagnostic interaction effects) was 13.0 percentage points. Insofar as the diagnostic moderation effect inherits the variance from unmeasured, ASD-related confounds, these confounds accounted for about 21% of the association between Verbal Language/Non-Verbal Communication and Social Withdrawal.

### Discussion

The demonstration of an undirected association between spoken and unspoken aspects of communicative ability (Verbal Language and Non-Verbal Communication) failed to falsify theoretical claims that variation in language function causes variation in social withdrawal symptoms. However, alternative explanations of this correlation are equally supported by these non-directional analyzes, including the possibility that degree of social withdrawal leads to altered language development (explicitly tested in Model 4, below). The DLD Diagnosis moderation effect in the ASD-Only cohort was small, disconfirming the notion that the mechanics of language development are different within autism classifications that require co-occurring DLD and those that include individuals with a broader range of language performance.

The association between Verbal Language/Non-Verbal Communication and Social Withdrawal held up in both the ASD-Only and ASD + Sibs cohorts. Because the association was seen in the ASD-Only cohort, we are safe from the possibility that the measured association is simply an artifact of other, unmeasured ASD-specific characteristics. The ASD-Diagnostic interaction term was about 21% of magnitude of the total effect, suggesting more variance in Social Withdrawal is explained by Verbal Language and Non-Verbal Confounds than by unmeasured, ASD-related confounds.

We are conscious of limitations of the Non-Verbal Communication construct in this convenience sample, which does not adequately parse the *ability* to produce gestures and other non-verbal behaviors from the *social intent* or *tendency* to generate these behaviors. This social intent may have conceptual overlap with the notions of social withdrawal and may thus overestimate the association between these two constructs. Within the diagnostic clinic and daily life of affected families, however, the combination of social/communicative intent and communicative ability combine to inform the social-communication diagnostic criterion for ASD (American Psychiatric Association, 2013).

Without further constraints in the model, the causal inference supported by these limited results is indeed open to circularity (Morton, 2005), *viz.*, that a non-directed statistical relationship between clinical aspects of a diagnosis and the presence of a diagnosis may be misinterpreted as evidence that those particular clinical aspects are *causative* of the range of behaviors encompassed in the diagnosis.

**Figure:**
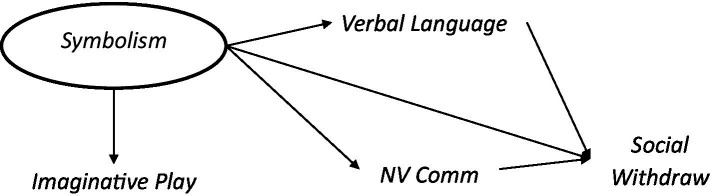


## Model 2: Dys-symbolic theory

### Introduction

Rutter’s initial proposals, tested above, invoked social withdrawal caused by frustration due to altered language *performance* and uncompensated for by non-verbal communication abilities. However, the literature evolved to hypothesize a central symbolic difference specific to autism and necessary not only for language performance but also for social engagement (Wing and Wing, 1971; Churchill, 1972; Ricks and Wing, 1976; Rutter, 1978). DLD children, in contrast to those with autism, demonstrated symbolic ability through intact non-verbal language (Rutter, 1978) and through imaginative play. Within the literature, imaginative play, verbal language and non-verbal communication were consistently discussed as manifestations of symbolic ability (Sheridan, 1969; Wing et al., 1977; Hermelin, 1978). The goal of this model was to examine the degree to which inter-individual differences in symbolic function could exist, as bounded by the shared variance among Verbal Language, Non-Verbal Communication and Imaginative Play constructs, and whether this latent factor is associated with Social Withdrawal.

### Methods

Three statistical inferences were needed to support—and each had the potential to falsify—key aspects of the causal model as proposed above. Firstly, for a latent factor representing symbolism to exist, there must exist joint variance shared among Verbal Language, Non-Verbal Communication and Imaginative Play variables. The presence of such a joint variance space does not guarantee that this variation validly represents Rutter’s, Rick’s and Wing’s notion of symbolism, but the absence of such a space would deal a sharp blow to the claim. Secondly, individual variability in this joint variance space must correlate with the value of Social Withdrawal. Thirdly, prediction of social withdrawal by Verbal Language, Non-Verbal Communication and Imaginative Play must by dominated by the joint variance space which we label “Symbolism,”^2^ and not from the marginal (non-“Symbolism”) variance of the Verbal Language and Non-Verbal Communication constructs.

To that end, we fit “Symbolism” as the dominant factor across Verbal Language, Non-Verbal Communication and Imaginative Play constructs, using a confirmatory factor analysis, independently in the ASD-Only ASD + Sibs cohorts.

Secondly, we used a GAM to estimate the association between this “Symbolism” factor and Social Withdrawal. Covariates and interaction effects were handled in the same as in Model 1.

Thirdly, we added the original Verbal Language and Non-Verbal Communication terms as predictors to the above models, separately in the ASD-Only cohort and in the ASD + Sibs cohort.

### Results

In the ASD-Only group, a joint space explained a good deal of variance among Verbal Language (*r*^2^ = 24.4%), Non-Verbal Communication (*r*^2^ = 52.2%) and Imaginative Play (*r*^2^ = 90.7%), which we labeled the “Symbolism” factor. The ASD + Sibs sample showed a similar latent construct (Verbal Language *r*^2^ = 39.4%; Non-Verbal Communication *r*^2^ = 73.1%; Imaginative Play *r*^2^ = 93.1%).

In the ASD-Only group, this “Symbolism” latent factor significantly accounted for 29.0% of the variance in Social Withdrawal beyond covariates alone, including both direct effects and those mediated by Verbal Language and Non-Verbal Communication. When we further added the unique effects of Verbal Language and Non-Verbal Communication (i.e., not originating with “Symbolism”), the variance explained in Social Withdrawal increased by a small degree, from 29.0 to 30.9%.

All interaction terms, including the DLD Diagnosis term, accounted for Δ*r*^2^ < 1%. General Intelligence interaction terms, added separately, increased the model *r*^2^ by 1 percentage point.

In the ASD + Sibs sample, the “Symbolism” factor significantly accounted for 60.2% of the variance in Social Withdrawal beyond covariates alone; adding the original Verbal Language and Non-Verbal Communication constructs increased the correlation (*r*^2^) only to 61.7%.

In ASD-Only samples, we observed a few statistically significant interaction terms, including the moderating role of DLD diagnosis, but the Δ*r*^2^ < 1% for all interaction terms, including General Intelligence. In the ASD + Sibs cohort, the ASD-Diagnostic moderating effect was significant (*p* < 10^−6^), and the *r*^2^ changed by 7.1 percentage points when the ASD-Diagnostic interaction terms were dropped. Insofar as the diagnostic moderation effect inherits the variance from unmeasured, diagnosis-related variables (confounders or other), these variables accounted for about an additional 10% of the association between “Symbolism” and Social Withdrawal.

### Discussion

In the absence of a direct and unambiguous measure of symbolism, it is impossible to know that the latent factor represented by shared variance space of Verbal Language, Non-Verbal Communication and Imaginative Play truly and specifically represents the original authors’ notion of symbolism. Nevertheless, the fact that there exists a latent factor with these properties, as predicted by theory, is non-trivial. Moreover, the fact that variance associated with this “Symbolism” latent factor explains most of the effect of communication constructs on Social Withdrawal is also non-trivially in line with theoretical predictions.

As in Model 1, however, these analyzes did not indicate the direction of the association.

These results also provide *soft* evidence to address concerns about the potential conceptual confounding of the Non-Verbal Communication construct with aspects of social engagement/withdrawal. Specifically, we believe that the Verbal Language construct does not suffer the same confounding (i.e., it veridically measures language ability without being confounded with social/communicative intent). The “Symbolism” construct is generated as the *shared* variance space of Verbal Language and Non-Verbal Communication (as well as Imaginative Play), and therefore is expected to contain less variance associated with social/communicative intent than is the original Non-Verbal Communication construct. If this social-intent-related variance contained in Non-Verbal Communication were large, we would expect that the association would be considerably stronger when Social Withdrawal is correlated with “Symbolism” plus the original Non-Verbal Communication construct than by “Symbolism” alone. This large increase was not observed (i.e., adding in both Non-Verbal Communication as well as Verbal Language increased *r*^2^ only by about 2 percentage points in each cohort).

## Model 3: “Coding”

### Introduction

Hermelin and O'Connor’s (1970) research demonstrated that autistic children have difficulty inferring the latent rules that underlie sequences and subsequently predicting the next element in a sequence (“coding”), whether those sequences are linguistic in nature or non-linguistic. They therefore viewed coding as a more fundamental function than symbolism—and one whose alteration in autism affects not only symbolic function, and therefore language production, but also non-linguistic phenomena as well (Hermelin, 1978). Quoting Hermelin (1978), “it would be an oversimplification to attribute the cognitive pathology in autism primarily to an impaired language system. According to our results, such an interpretation fails to take account of the restricted access which autistic children seem to have also to other, nonlinguistic representations.”

**Figure:**
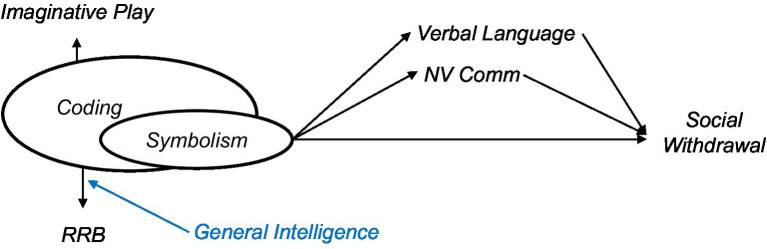


Our dataset does not include a direct experimental measurement of coding, such as in Hermelin and O'Connor (1970), but we can make some progress if we include Hermelin and O'Connor’s (1970) auxiliary premise that RRB is a secondary attempt to simplify the inputs from the physical world because of this alteration in coding/prediction. Rutter actively dismissed the non-linguistic implications of Hermelin and O’Connor’s coding findings, suggesting that the non-linguistic manifestations were confounded by “one important limitation and that is that in many of their studies the children [had ID]” (Rutter, 1978). This counter-claim was a bit tenuous on its face, even at the time of its initial proposal, given Hermelin and O’Connor’s ID control group, but for completeness’ sake, we test it here.

### Methods

We attempted to answer two questions: whether symbolism/coding (again operationalized as joint variance space of Verbal Language, Non-Verbal Communication and Imaginative Play) also predicted RRB (consistent with the view of Hermelin and O’Connor) and whether any relationship between symbolism/coding and RRB was obliterated by adjusting for General Intelligence (consistent with the response of Rutter).

To that end, we used RRB as the outcome variable within a GAM, with the “Symbolism” factor as the predictor, as contrasted with the covariates-only model. Our test to determine whether general intelligence explained RRB better than coding/symbolism was to compare the original model with one adjusting for the General Intelligence construct. Note that different versions of the RRB constructs and General Intelligence constructs were used in the ASD-Only models vs. ASD + Sibs models.

### Results

15.4% of participants in the ASD cohort reported diagnoses of ID, As Did 1.4% of sibs.

In the ASD-Only cohort, there was a significant association between “Symbolism” and RRB (*r*^2^ = 2.1%), after adjusting for covariates. After adjusting for General Intelligence, there was no significant decrease in the association between “Symbolism” and RRB, and the effect curve between these two constructs did not change significantly before and after adjusting for General Intelligence.

All interaction effects (including those associated with DLD) contributed 1.26% of variance, while the Parent Education × Sex interaction term is the only one that is statistically significant.

In the ASD + Sibs cohort, the model again demonstrated a significant link between “Symbolism” and RRB (*r*^2^ = 38.6%), after adjusting for covariates. After adjusting for General intelligence, there was no decrease in the magnitude of the association between “Symbolism” and RRB. Additionally, the effect curve between “Symbolism” and RRB did not change significantly before vs. after adjusting for General Intelligence (meaning the effect of “Symbolism” → RRB cannot be explained by the confounding of General Intelligence).

All significant interaction terms contributed <1 percentage point of effect size.

The moderating effect of ASD Diagnosis was again significant (*p* < 10^−6^), but only contributed <1 percentage point to the model *r*^2^.

### Discussion

In summary, the association, albeit modest, between our “Symbolism” factor and RRB supports (or fails to falsify) Hermelin and O’Connor’s claim that there exists a coding capacity that serves both the symbolic nature of language and also non-linguistic features of autism. Rutter’s claim that the non-linguistic/non-social features of autism are associated with ID rather than being a consequence of the same construct that supports the symbolic function of language is undermined by the statistical inability of General Intelligence to explain the empirical relationship between “Symbolism” and RRB.

The strength of the conclusions is supported not only by similar results from two samples in this analysis, but also by two different sets of items to measure both General Intelligence and RRB in the different cohorts. In light of anecdotal concerns about covariance in caregiver-report items and issues with recall bias (affecting the items in the ASD + Sibs version of the General Intelligence construct), it would be preferable to have the results of IQ testing performed by qualified psychometricians. Doing so would allow us to determine whether the IQ distribution in our sample was skewed and to determine parametrically whether there were different relationships between IQ and RRB at different ranges of the IQ dimension (Note that for statistical testing purposes, our non-linear models accounted for this possibility.)

## Model 4: Dyscultural model

### Introduction

The first model, and its elaborations in Models 2 and 3, focused on the role of impaired communication (and its cognitive bases) as causing social withdrawal in ASD. Alternative accounts posited that social withdrawal is primary and subsequently causes impairments in language development [Bosch, 1962/1970; Richter, 1978; also discussed in Rutter (1978)].

We know today that differences in social attention can affect rates of language development in children generally (Boucher, 2011a). We further know that language exposure can affect verbal development (Hoff and Tian, 2005), and the social withdrawal observed in some autistic children often engenders less expressive language from others (Wozniak et al., 2017).

**Figure:**
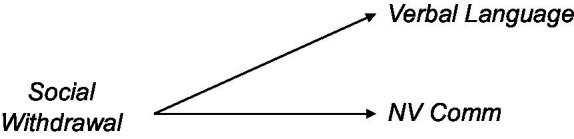


### Methods

The goal for this model contrast was to determine causal directionality between social withdrawal on the one hand and verbal language/nonverbal communication on the other. To this end, we took advantage of the concept of conditional independence (Waldmann and Martignon, 1998). It works on the following reasoning: if inter-individual variability in a single cause (here, Social Withdrawal) results in inter-individual variability in an effect, then the values of the cause and effect variables will correlate with each other across participants. If the cause results in two effects (here, Non-Verbal Communication and Verbal Language), then the cause variable will correlate with each of the effect variables. Moreover, because effect_1_ and effect_2_ each correlates with the cause variable, then effect_1_ and effect_2_ will necessarily correlate with each other. Finally, because the relationship between effect_1_ and effect_2_ is due (at least to some degree) to the shared variance inherited from the cause variable, if one partials out the value of the cause variable from the effect_1_-effect_2_ correlation, then the effect size of the effect_1_-effect_2_ correlation would necessarily decrease.

### Results

In the ASD-Only sample, the Pearson’s correlation between Verbal Language and Non-Verbal Communication (covaried for Age, Sex, Parental Education, and Family Type) dropped from *r* = 0.085 (*p* < 10^−9^) to 0.031 (*p* = 0.025) when adjusted for Social Withdrawal for children diagnosed with DLD. In children not diagnosed with DLD, the drop was from 0.086 (*p* < 10^−9^) to 0.025 (*p* = 0.06, not statistically significant) when conditioning on Social Withdrawal. In both sub-cohorts, the drop in effect size and increase in *value of p* indicates that verbal language and non-verbal communication cannot be a common *cause* of social withdrawal.

In the ASD + Sibs sample, we saw a drop from *r* = 0.145 (*p* < 10^−10^) to *r* = 0.057 (*p* < 10^−10^). Although this drop in effect size suggests that it is most likely that the communication→social withdrawal link does not obtain, the *value of p* remained small.

### Discussion

The use of a test of conditional independence allowed us to evaluate the direction of the association between social engagement and communicative function. Contrary to the language-primary theories of ASD, the results in the ASD-Only group are inconsistent with the notion that variability in verbal language and non-verbal communication are common causes of social withdrawal. The results of the ASD + Sibs cohort did not meet the strict criterion by which the association between Verbal Language and Non-Verbal Communication would have been reduced to non-significance once Social Withdrawal was adjusted for, though the effect size (*r*^2^) did show a numerical decrease by a factor of more than five.

While the graph holding that social withdrawal affects the development of verbal language and non-verbal communication is supported by this analysis, other graphs are equally well supported, including non-verbal communication being the primary alteration (Mundy et al., 1986, 1990). Additional observations with a greater number of constructs or even interventions may be needed to decide among these remaining possibilities.

## General discussion

### Substantive conclusions about language-primary theories of autism and alternative accounts

When we synthesize the results of all our models, two substantive conclusions regarding the language theories of autism remain, one relating to the direction of the causal association between social withdrawal and communicative performance, and one relating to the existence of a symbolism/coding construct and its association with social withdrawal and RRB. Regarding the first conclusion, the evidence from causal modeling of SPARK data makes it unlikely that variation in language and non-verbal communicative ability unidirectionally influences social withdrawal/engagement (Rutter, 1966, 1968; Wing and Wing, 1971). The claim that social withdrawal affects language development and non-verbal communication (Bosch, 1962/1970; Richter, 1978) is supported by the results, but we should recognize that equally supported are graphs in which, for example, non-verbal communication → social engagement/withdrawal → verbal language [*c.f.* Mundy et al., 1986)]. More complex interactions, such as a feedback loop (Wozniak et al., 2017), also cannot be resolved by the current methods. Because “any finite set of data can be explained by any of an infinite number of equations,” our strongest conclusion is that the results are *inconsistent* with and help falsify the accounts that hold language as a primary influence on social withdrawal, such as expounded by Rutter. Insofar as our conclusions are convergent with the actual evolution of the literature, Rutter himself later reversed his position and later endorsed the conclusion we have discovered here: that it is possible that autism-associated language differences may be a consequence from primary-social mechanisms [Rutter, 1983; see also (Boucher, 1976)]. Language continues to be studied in autism, but not as a primary cause of a wide range of symptoms (Iverson and Goldin-Meadow, 2005; Landa and Goldberg, 2005; Eigsti et al., 2011; Luyster et al., 2011). If anything, language features of autism have in the last few decades been treated as explananda (“those things which must be explained,” i.e., the causal outcome of other factors) of Theory of Mind, Executive Function and Weak Central Coherence causal theories.

Our second substantive conclusion revolves around the estimate of a factor statistically comprised of a joint variance space inherited from Imaginative Play, Non-Verbal Communication, and Verbal Language, and one which also correlates with Social Withdrawal and RRB. Although the results from Model 4 negate the original directional claims in Models 1–3, nevertheless we still have statistical evidence for the existence of such a factor. Because of its association with RRB (albeit a small one), we are closer to the truth if we follow Hermelin and O’Connor by labeling it “Coding” rather than “Symbolism.” (Here, too, Rutter changed his position (Rutter, 1983).) However, without a direct test of coding [e.g., as measured experimentally by Hermelin and O'Connor (1970)], we still cannot assign this label with any confidence. Nevertheless, the presence of this statistically defined construct and its relationship to social withdrawal and RRB gives us an affordance for further hypothesizing and testing. There are more trivial possibilities represented by this joint variance space: that it reflects general task engagement [e.g., Rimland, 1964 as a claim of underarousal and under-engagement] or reporting bias associated with the questionnaire methodology (anecdotally discussed among psychometricians). Using diverse methods of measurement reduces the susceptibility to such biases and confounds, which could either over-or under-inflate estimates of relationships between constructs. The advantage of a theoretically informed approach over a purely empirical approach is that theoretical constructs are defined in such a way as to allow fusion of data from multiple modalities, participant samples and experimental designs, each with differential sensitivity to artifact (Cronbach and Meehl, 1955; Andreas, 2017; Pearl and Bareinboim, 2018; Ewen et al., 2021).

A further possibility with regard to our empirically estimated “Coding” variable is that it reflects a summary quantitative autism trait (Constantino, 2011), though we view this possibility as being less likely because it has only a modest correlation with diagnostically essential RRB features. We acknowledge that within other psychological and cognitive neuroscientific study of psychiatric conditions, statistical adjustment for a condition-specific “generalized neurocognitive impairment” explained the bulk of variance in clinical performance and reduces statistical associations between individual constructs (Reilly and Sweeney, 2014; Reilly et al., 2017); these results from other conditions are consistent with empirical evidence of a unidimensional, quantitative autism trait (Constantino, 2011; though see empirical results supporting a competing model (Happé et al., 2006; Happé and Ronald, 2008; Robinson et al., 2012)). However, given the rather modest correlation between some features (e.g., “Symbolism” and RRB), it would appear that the effects we are considering here correspond to the inter-individual heterogeneity Constantino and colleagues refer to as “sitting on top of the substructure” reflected by the core, unidimensional, quantitative autism trait (Constantino et al., 2021).

We should note that the theories as stated in the original literature also included additional elements for which our convenience sample did not have items, including the distinction between receptive vs. expressive language, and pragmatic language usage. With more richly specified models, constructs parsed to an optimal level of granularity, and specific measures, we can provide models that have a wider explanatory scope, more effectively avoid circularity and are more amenable to falsification.

### Substantive conclusions about age and role of development

We saw no substantial evidence of a moderating effect of Age in any of our models. Models 1 and 4 are “developmental” in the sense that performance of an ability at a certain level, in a way that interacts with the environment, leads to changes in another ability (e.g., social withdrawal causes an alteration in the typical environmental-dependent learning of language). This form of causal influence manifests as a lag between observable behavior from the causal cognitive construct and the consequent observable performance of the downstream cognitive construct. If some intervention were to directly minimize a child’s social withdrawal today, we would predict that the consequent expansion of language abilities would be measurable after a period of social engagement, language learning and language practice. The least ambiguous way to measure this lag effect would be through collection and analysis of longitudinal data. Longitudinal data incorporate temporal precedence that makes the conclusion of causality more dependent on the structure of the data and less dependent on model assumptions.

However, even in this cross-sectional dataset, we did not find evidence of Age moderation with a relevant effect size in any of analyzes. If our theories entail a lag between the manifestation of the cause and the manifestation of the effect, then we would predict the presence of a substantial interaction effect, which we did not find.

Our results are further susceptible to the possibility of critical periods, *viz.*, that our data collection did not occur over the age range at which irreversible developmental effects occur. Rutter specifically denied that critical periods were relevant to his theoretical accounts (Rutter, 1968), however research since that time has endorsed the relevance of critical periods to development; in the case of autism specifically, *early* intervention appears to have the best chance at enhancing adaptive outcomes (Tonge et al., 2014).

### Sampling and diagnostic moderation effects

As noted earlier, descriptive effects mis-labeled as causal claims are subject to fallacious circular reasoning (e.g., the fact that autistic individuals have differences in language serves as evidence that language differences *cause* autism…which in turn is characterized in part by language differences; Morton, 2005). This circularity highlights the inferential challenges associated with deriving causal evidence from case–control data, particularly where case status is defined by heterogeneous categorical psychiatric diagnoses [e.g., *via* the Diagnostic and Statistical Manual of Mental Disorder (DSM) (American Psychiatric Association, 2013)]. Moreover, as the heterogeneity of the autism spectrum (Wing, 1988b) or constellation (Fletcher-Watson and Happé, 2019) is possibly expanding due to changing diagnostic thresholds (Wing, 1976b), we recognize that individuals can meet diagnostic criteria for autism through a variety of symptom patterns (DeMyer et al., 1971; Wing and Gould, 1979; Wing, 1988a). To wit, autism diagnosis was treated as a proxy for social withdrawal by the authors whose theories motivated our analyzes, whereas we now recognize that not all autistic individuals demonstrate social withdrawal (Wing and Gould, 1979). For those reasons, our primary approach was within-group (ASD-Only analyzes), using targeted behavioral explananda (e.g., language differences cause social withdrawal) rather than diagnostic explananda (e.g., language differences cause autism). Moreover, we have far greater statistical power when we quantify each individual’s expression of a particular behavioral explanandum rather than lumping together individuals who show high levels of that feature with those who do not.

Nevertheless, even making the explanandum as focused as possible still leaves statistical conclusions and subsequent inference sensitive to biases and confounds associated with suboptimal sampling. The theories we have evaluated were developed within a conception of autism that included language differences as an essential criterion, and the SPARK data were sampled under a more inclusive diagnostic framework. We contended with known and suspected language and IQ differences between the sample and the theoretical population by evaluating the moderating effect of General Intelligence and DLD diagnosis (both of which showed a small effect sizes in all analyzes), but there may be other, unmeasured differences between individuals Rutter and Hermelin studied and those sampled in the current diagnostic era.

Future efforts to study feature-to-feature, rather than feature-to-diagnosis associations, while “ignoring” the remainder of the (non-independently distributed) patient characteristics that contribute a DSM syndromic diagnosis, has limitations. Within these sparse models, failing to account for all other cognitive and behavioral features of a syndrome leads to a substantial risk of confounding by those features. For that reason, we also evaluated an ASD + Sibs cohort and specifically accounted for diagnostic interaction effects within that cohort. We viewed the presence of a diagnostic moderation effect as representing a placeholder, not unlike dark matter, for effects that were only indirectly observed and not explicitly modeled. In Models 1 and 2, this diagnostic interaction term explained 7–13 percentage points of the variance in the targeted relationships. In future work, we may see how much of the diagnostic interaction term is explained away by a unidimensional, latent quantitative autism trait (Constantino, 2011). However, the assessment of a latent autism trait is only meaningful when a sufficiently broad scope of autistic features is sampled.

Sampling approaches are also highly relevant to ensuring that data are representative as a whole, and the Simons Foundation is taking proactive steps to increase diversity within the SPARK sample (Duhon et al., 2022).

The literature in the 1960’s and 1970’s was heavily invested in the extensive use of alternate clinical control groups for focusing in on how autism may be similar or different to those other conditions (Yule, 1978). Autism was the relatively new diagnosis, and far more was known at the time about the psychology of sensory impairments, DLD and ID. As autism research developed its own momentum, much of the work proceeded over subsequent decades in a case–control mode. Skip ahead to recent times and increasing calls for “reversion” to trans-diagnostic approaches (Happé and Frith, 2006; Constantino et al., 2021). Trans-diagnostic research is particularly critical in autism research, as which many of the conditions on the differential diagnosis list are also highly co-occurring with ASD. Rather than trapping ourselves in a situation where we force ourselves to attribute a behavior either to autism or to co-occurring Attention-Deficit/Hyperactivity Disorder (ADHD) (e.g., as an independent variable within a case–control study), we can merely quantify the behavioral explanandum and evaluate its association with a particular mechanism. By formalizing and statistically evaluating causal models with behavioral (rather than diagnostic-category) explananda, we may in this way sample from multiple current-day diagnoses and conjointly model behavioral explanantia and proposed mechanisms that are relevant to one or both diagnoses (Harris, 2016).

Within the 1980’s to early 2000’s Developmental Psychology era of autism research, which was focused on the relationship among Executive Function, Weak Central Coherence and Theory of Mind accounts of autism, the field paid considerable attention to whether certain cognitive differences (somewhat arbitrarily thresholded and binarized) were uniquely and universally present (only) in autistic individuals (whose diagnostic status is itself some sort of semi-arbitrary binarization of multidimensional behavioral characteristics; Rajendran and Mitchell, 2007). The approach used here, by contrast, focuses not on binary-binary associations of mechanism and diagnosis, but on continuous-continuous relationships of cognitive mechanism and behavioral explanandum, effectively dissolving questions of uniqueness/universality and replacing them with quantitative expressions of *how much* a certain mechanism contributes to a certain behavior.

Again, DSM diagnosis cannot be completely ignored however, both because of the potential confounds of non-independently-distributed, unmodeled features of the syndrome, but also because of practical considerations around participant recruitment. DSM diagnoses provide a method of identification for study recruitment, and the trans-diagnostic approach supported here and by others (Happé and Frith, 2006; Constantino et al., 2021) requires recruitment of multiple diagnoses. Conditions that mimic autism, share at least some behavioral features, co-occur at higher-than-population rates (Ewen and Shapiro, 2005) or provide important contrasts include DLD, ID, ADHD plus language delay, anxiety plus language delay, Sensory Processing Disorder (without other diagnostic aspects of the autism phenotype), selective mutism, social anxiety, Obsessive Compulsive Disorder, stereotypic movement disorder, “quirky” phenotypes, shyness, introversion, chronic distraction (e.g., from chronic pain), Social (Pragmatic) Communication Disorder (i.e., without RRB), isolated developmental prosopagnosia, isolated alexithymia, sensory impairment, Developmental Coordination Disorder/dyspraxia, epilepsy (social features), TBI (social features), the now-defunct Non-Verbal Learning Disorder, features of Frontal-Temporal Dementia, psychotic disorders, Major Depressive Disorder, Adjustment Disorder, Tourette Syndrome, and Schizoid/Schizotypal Personality Disorder. We can also enhance the generalizability of our understanding by increasing the diversity of our autism and neurotypical samples, particularly over ever-evolving diagnostic criteria and thresholds (Rosen et al., 2021). We may study different levels of severity, intermediate phenotypes (DeMyer et al., 1971; Wing, 1976b; Rutter, 1978; Landa et al., 1991, 1992; Pickles et al., 2000; Dawson et al., 2002), syndromic and non-syndromic etiologies, and different behavioral presentations within ASD (Wing and Gould, 1979; Wing, 1988a; Mottron and Bzdok, 2020) data-driven methods of hierarchically modeling clinical features across the neurodiversity spectrum (Clementz et al., 2016; Jacobs et al., 2021; Scheerer et al., 2022). Such trans-diagnostic work is already paying dividends at the genetic level (Lord et al., 2020) and may again do so at the cognitive level.

In the future, we may also define our “diagnostic” moderation terms not based on current behavioral syndromes, such as those defined by DSM, but by empirically derived clusters derived from data at different levels of analysis, such as the genetic level (Torres, 2021), the “imaging” level (Clementz et al., 2016) or *via* modern artificial intelligence approaches that capture delimited clusters of behavioral performance (Mottron and Bzdok, 2020). Trans-diagnostic clustering based on fine-granularity cognitive research as well as data from other levels of analysis may redefine our nosologies (Ewen et al., 2021).

In summary, both fully categorical (e.g., DSM diagnoses) and fully dimensional (quantitative behavioral explananda) approaches have their limitations. However, we can discard the notion that “the complex and heterogeneous ASD phenotype will be explained by a perfectly unique and universal cognitive cause” by modeling multiple candidate mechanisms conjointly, consistent with the approach of so-called Multiple Deficit accounts of autism (Wing and Wing, 1971; Goodman, 1989; Happé and Ronald, 2008; Boucher, 2011b). By studying a diverse range of the autism spectrum as well as current diagnoses that show some overlapping features, we will be able to assess the magnitude of contribution of a diversity of mechanisms, some related to only one existing diagnosis, and others relevant to multiple.

### Generating and refining theories

To this point, we have talked about the evaluation of theories. But what constraints exist in the development of theories and their explication? Keeping theories as the center of our efforts is important not just to raise the bar on statistical tests, but because theory is our primary unit of scientific understanding. Theory is what allows us to link, for example, language as measured by a psychoeducational test with language as expressed in academic performance with language as employed in social interactions (Cronbach and Meehl, 1955; Andreas, 2017). And it also allows us to constrain the relationships we enter into our theories and causal graphs (e.g., it is meaningful to talk about an alteration in symbolic function causing a difference in language function but not the reverse relationship, as symbolic cognition must logically precede its expression *via* language). That is to say that the theories investigators propose in the first instance are naturally bound by existing knowledge from a wide variety of sources (van Rooij and Baggio, 2021). In the case of autism, novel theorizing was in many instances explicitly guided by existing knowledge around typical language development, DLD, sensory impairments and ID. That is to say that theorizing about autism is constrained from the start by existing knowledge outside the realm of autism research. Writing about the philosophy of science as related to physics, Nagel (Nagel, 1979), posed each theory of a specific phenomenon (e.g., the behavioral phenotype of autism) as a “sub-theory” of a unified theory addressing all phenomena within a broader scientific discipline (e.g., Developmental Psychology or Cognitive Psychology).

So, the notion of conjointly modeling theories of multiple current diagnoses and sampling from those populations is not only of practical value but is also important in developing the “grand” model of a particular subfield of psychology that would explain variance across “typical development” as well as all relevant clinical conditions. Within the current work, we have seen examples of different ways in which distinct theoretical accounts can relate to each other. Two theories may be mutually exclusive (e.g., social withdrawal→language vs. *s*ocial withdrawal←language), one may supervene on another (e.g., coding explains the phenomena attributed to symbolism, and also explains RRB while symbolism alone does not), they may explain different explananda, or they may both contribute (additively or supra-additively) to the same explanandum.

In order to model theories conjointly, we first need to explicate theories well. The theories formalized here as causal statistical graphs were “verbal” and “imprecise” (Fried, 2020). The lax specification of theories has been a significant hinderance to theory-evaluating work (Morton, 2005). Computational models, again gaining popularity (Devezer et al., 2021; van Rooij and Baggio, 2021), may contribute to formalization of theories, but even verbally-stated theories can be held to the standard of providing precise predictions and explicating their areas of divergence from other accounts (Fried, 2020). In the work presented here, we found that reading the evolution of the literature over decades, particularly as the various authors responded to each other, helped add specificity to models that may not have been present in a lax summary of claims, handed down through a “game of telephone.”

However, even after theories are formalized, it may not be trivial to model one conjointly with another. Autism research has attracted investigators from a wide range of disciplines/subfields of psychological science—Kraepalin Psychiatry, Psychoanalysis, Clinical Neurology, Behavioral Psychology, Cognitive Psychology, Neuropsychology/Behavioral Neurology, Piagetian and Connectivist Developmental Psychology, Cognitive Neuroscience, and, more recently, computational approaches to psychology, such as Predictive Coding and Computational Motor Control—each with its own vocabulary, set of constructs (ontology) and premises. Bringing these constructs and frameworks in alignment in a way that can be put to empirical testing is non-trivial and has been the effort of both formal (Westmeyer, 1989) and informal (Allport, 1955) types of efforts within psychology broadly. Perhaps the time is ripe for such an effort within autism research.

## Conclusion

We present evidence against the language-primary theory of autism in the form originally proposed by Rutter. Our results support (or fail to falsify) primary-social theories (Richter, 1978) or subsequent accounts which hold a primary role for non-verbal communication (Mundy et al., 1986). We also provide evidence for a construct defined statistically as the shared variance space of Verbal Language, Non-Verbal Communication and Imaginative Play; this construct also correlates with Social Withdrawal and RRB. It has the statistical features predicted by Hermelin and O’Connor’s coding construct, but without a direct test of coding ability, it is impossible to know whether that is what it is.

This “test flight” of using large datasets and network analyzes to test theory has also highlighted principles for a broader research agenda. First is the principle of *specification* (and constraint) of our analyzes *a priori*; this is accomplished in the first instance by formalizing theories (Morton, 2005) as directed graphs, and assessing the pre-existing observations and assumptions on which the theory was based (van Rooij and Baggio, 2021), whether inherited from autism research or from other domains of psychological research. We further constrain the graphs by including as many constructs as warranted (Peirce, 1960; Rosenbaum, 2017); the greater the number of predicted relationships, the easier to falsify or test competitively with other theories. Measurement of constructs though multiple methods (Ewen et al., 2021) and with adequate controls helps eliminate sensitivity to artifacts that can either over-or under-estimate statistical relationships; the use of theoretical constructs within graphs rather than solely observable variables allows us to fuse information from multiple modalities (Cronbach and Meehl, 1955; Andreas, 2017; Pearl and Bareinboim, 2018). Longitudinal data further help constrain interpretation in developmental contexts.

The second principle is *quantification*. The reliance on binarization—of presence/absence of diagnosis, of presence/absence of a “candidate cause” cognitive difference—has led to concerns about whether a single cognitive difference is both necessary and sufficient to explain the rich and heterogeneous behavioral phenotype of autism. However, considering the *weighted* contribution of explanatory mechanisms to explananda dissolves these concerns, particularly where we suspect that there may be multiple mechanisms working in parallel or in interactive fashion (Goodman, 1989; Happé et al., 2006). Also, quantification of behavioral features (rather than contrasting results over multiple case–control studies, in which diagnosis serves as the explanandum), renders results more resistant to changing definitions of autism and to sampling biases.

The third principle is *integration,* across existing diagnoses (Happé et al., 2006; Happé and Ronald, 2008) and across theories (theories about autism and theories about other diagnostic groups). By focusing on behavioral (rather than diagnostic/syndromic) explananda, we can model together data from multiple conditions that have some relevance to autism. Testing in integrative fashion, we define whether different theories relate to each other as additive/super-additive contributors to the same explanandum, as parallel contributors to different explananda, in supervenience or are truly mutually exclusivity. To do so well, we must be aware of the need for formal explication of theories (and their predictions), as well as challenges inherent in integrating theories across psychological disciplines. But in achieving this integration, we begin to build up models that span typical and diverse development and inter-individual differences in performance. Recent works bringing together multiple theories of autism in one place are tremendously helpful in integrating theories (Rajendran and Mitchell, 2007; Fletcher-Watson and Happé, 2019).

We are conscious that the approaches suggested here will inherently require very large samples. However, smaller-scale but more directed data collection will also be an important part of defining particular relationships that are important for constraining our models.

What is the end-goal of this program of research? One of the authors (JBE) is a Neurodevelopmental Disabilities physician, and the key cognitive task in clinic with complex patients is to create a mental model *effectively equivalent to those studied here,* which relates multiple cognitive factors to multiple behavioral features to participation in the community. With an adequate mental model, the clinician can most efficiently and effectively propose interventions (clinical or environmental; Woodward, 2003) that will alleviate points of friction that affect adaptive function and quality of life. In so far as any disability reflects a mismatch among an individual’s cognitive profile, social supports and social expectations, we are conscious of the fact that the most apt explananda, which are causally downstream even of behavior, may be these functional (community participation) and quality-of-life outcomes.

## Data availability statement

Publicly available datasets were analyzed in this study. This data were provided by the Simons Foundation Autism Research Initiative; https://www.sfari.org/resources. R code for the analyses performed can be obtained from https://github.com/bhtang127/SPARK-Language-Withdrawal.

## Ethics statement

The studies involving human participants were reviewed and approved by the Western IRB, Inc. Written informed consent to participate in this study was provided by the participants’ legal guardian/next of kin. Review of de-identified data was reviewed by the Johns Hopkins Medicine IRB and was determined not to be human subjects research.

## Author contributions

BT designed optimal approaches for statistical analysis, implemented the analyzes, and edited the manuscript. ML assisted in the review of theoretical models and items for constructs. JA assisted in the review of theoretical models and items for constructs. EW assisted in the review of theoretical models and items for constructs, obtained the dataset, advised on optimal cleaning of data, and edited the manuscript. BC supervised the statistical approaches and interpretation of the data and edited the manuscript. JE obtained funding, conceptualized the research, performed the literature review, helped obtain the dataset, selected items for constructs, explicated the models, interpreted the data, and wrote the manuscript. All authors contributed to the article and approved the submitted version.

## Funding

This work was supported by R01 MH113652 (to JE) and P50 HD103538.

## Conflict of interest

The authors declare that the research was conducted in the absence of any commercial or financial relationships that could be construed as a potential conflict of interest.

## Publisher’s note

All claims expressed in this article are solely those of the authors and do not necessarily represent those of their affiliated organizations, or those of the publisher, the editors and the reviewers. Any product that may be evaluated in this article, or claim that may be made by its manufacturer, is not guaranteed or endorsed by the publisher.

## Footnotes

^1^We use lower-case labels for abstract constructs (e.g., social withdrawal) and upper-case labels for the variables used to quantify those constructs in our models (e.g., Social Withdrawal, Age).

^2^We use scare quotes in speaking about "Symbolism" because this factor is defined solely in statistical terms as a joint variance space shared from other constructs, and not based on specific items that get to the conceptual construct of symbolism, as we have with these other constructs. We consider below that there may other conceptual constructs, apart from symbolism, that could also explain this joint variance space.

## Abbreviations

ADHD: Attention-Deficit/Hyperactivity Disorder
ASD: Autism Spectrum Disorder;
DCDQ: Developmental Coordination Disorder Questionnaire
DLD: Developmental Language Disorder
DSM: Developmental and Statistical Manual of Mental Disorders
GAM: Generalized Additive Model
ID: Intellectual Disability
IQ: Intelligence Quotient;
LKS: Landau–Kleffner syndrome
MIRT: Multivariable Item Response Theory
PCA: Principal Components Analysis
RCT: Randomized Controlled Trial
RMSEA: root mean square of error approximation
RRB: Restricted and repetitive behaviors and interests
SCQ: Social Communication Questionnaire
SPARK: Simons Powering Autism Research
SRMR: standardized root mean square residuals
TD: Typically developing.
